# The Efficacy of Bisphosphonate in the Treatment of Giant Cell Tumour of the Bone: A Systematic Review and Meta-Analysis

**DOI:** 10.5704/MOJ.2303.012

**Published:** 2023-03

**Authors:** MF Deslivia, SD Savio, IGE Wiratnaya, P Astawa, KS Sandiwidayat, NG Bimantara

**Affiliations:** Department of Orthopaedics and Traumatology, Universitas Udayana, Denpasar, Indonesia

**Keywords:** bisphosphonate, giant cell tumour, meta-analysis

## Abstract

**Introduction:**

Anti-osteoclastic mechanism of Bisphosphonate (BP) is crucial to treat Giant Cell Tumour of the Bone (GCTB), however no established guidelines of its use have been published. This systematic review and meta-analysis is the first to summarise recent clinical studies on the subject.

**Materials and methods:**

A systematic search was performed based on PRISMA guidelines for clinical trials of BP administration in GCTB. Baseline data including BP regimen, dose and timing was summarised. The primary outcomes assessed were recurrence rate, metastases, survival rate, functional outcome, clinical outcome, radiological outcome, and adverse effect.

**Results:**

We identified 8 articles from 2008-2020. Most studies administer 4mg of Zoledronic acid post-operatively, with five studies mentioning pre-operative administration and six studies describing post-operative administration. There was a total of 181 GCTB cases analysed in this study. The BP group presented lower recurrence rate than control group (three studies; Odds Ratio [OR] 0.15; 95% Confidence Interval [CI], 0.05 – 0.43; p<0.05; heterogeneity, I2=0%). As for survival rate, BP group is comparable to control group (two studies; OR 1.67; 95% CI, 0.06 – 48.46; p=0.77; heterogeneity, I2=65%).

**Conclusion:**

Bisphosphonate therapy offers satisfactory recurrence rate, functional outcome, clinical outcome, radiological outcome, survival rate and metastases rate in patients with GCTB, with minimal adverse effects. Pre- and post-operative administration of bisphosphonates in combination might be the most beneficial in minimalising the recurrence rate.

## Introduction

Bisphosphonate (BP) application in Giant Cell Tumour of the Bone (GCTB) as systemic adjuvant treatment is still controversial. In vitro and animal studies showed its effect in inducing stromal cell inhibition, apoptosis and osteogenic differentiation. However, studies with high level of evidence are still needed to strengthen the recommendation of routine BP application in GCTB treatment. This systematic review and meta-analysis is the first to summarise recent clinical studies on the subject.

The mainstay of treatment for Giant Cell Tumour of the Bone (GCTB) is surgical resection, either en bloc resection or curettage, with or without local adjuvants. However, high risk of recurrences after this primary treatment creates the need for systemic adjuvant treatment such as BP. As part of a reliable treatment regimen for osteoporosis, metastatic bone disease, and Paget’s disease, the use of BP for GCTB and its efficacy has not been discussed a lot in literature. As an analogue of pyrophosphate, BP with its anti-osteoclastic actions seems to be promising in improving the outcomes of patients with resectable as well as unresectable GCTB^1^.

There are still no established guidelines on the indication, dose, regimen, and efficacy of BP for patients with GCTB. Most trials administer BP as a part of post-operative treatment, yet pre-operative application is an interesting subject to explore, where it has the capability of reducing tumour size before surgery. This provides the basis for the study question: what is the efficacy of bisphosphonate therapy for GCTB, and what is the proper dosing and time of administration? Through this systematic review, we aim to discuss the details of BP treatment in previous studies, while the meta-analysis part will summarise quantitative evidence of BP effect in terms of survival and recurrence rate.

## Materials and Methods

**Fig. 1:**
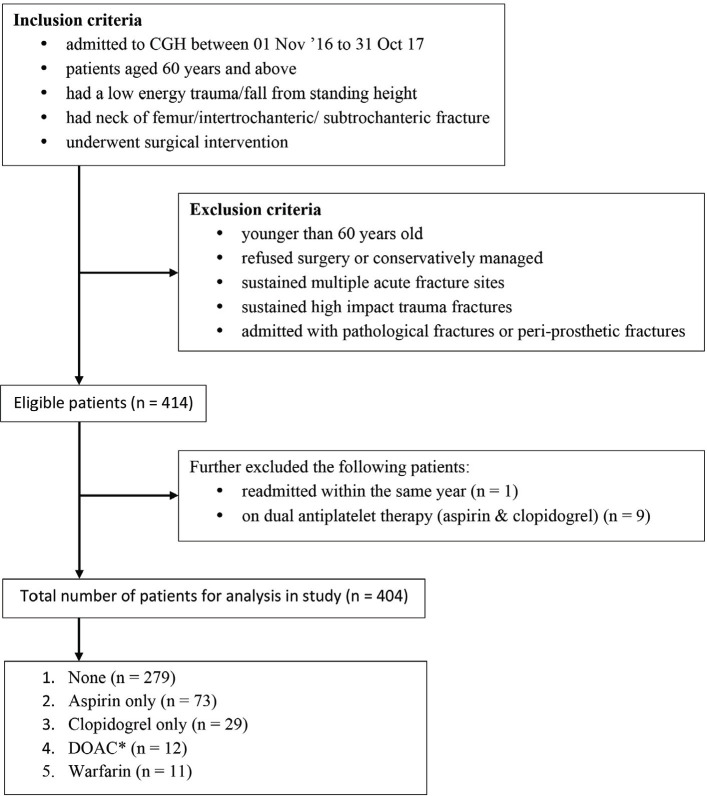
Flow chart showing article selection.

This systematic review was performed in accordance to Preferred Reporting Items for Systematic Reviews and Meta-analysis (PRISMA) guidelines (Fig. 1). We performed systematic search through MEDLINE, Embase, Cumulative Index to Nursing and Allied Health Literature (CINAHL), Web of Science, Cochrane Central Register of Controlled Trials (CENTRAL), and ClinicalTrials.gov. Language was limited to English. Our search strategy was as follows: keywords such as “Giant Cell Tumour of Bone” and “Bisphosphonate” and “Outcome” were used. Those data were then manually scanned and reviewed by all authors based on inclusion and exclusion criteria according to PICO (Population, Intervention, Comparison, Outcome) as depicted in Table I.

**Table I:** Population Intervention Comparison Outcome (PICO) table describing inclusion and exclusion criteria.

Study Component	Inclusion	Exclusion
Population	Giant Cell Tumour of the bone in any location Any age Recurrent, metastatic, pathological fractures Any pathological stage	Less than 3 months of follow-up Animal studies
Intervention and Comparison	Bisphosphonate Treatment Control group Noncomparative studies Accompanied or not accompanied by surgical interventions	All other treatments
Outcome	Recurrence rate Metastases Survival rate Functional Outcome Clinical Outcome Radiological Outcome Adverse Effect	No outcome mentioned or different outcomes
Publication	Primary research published in English in a peer-reviewed journal	Abstracts, editorials, letters Duplicate publications of the same study that do not report on different outcomes Conference presentations or proceedings
Design	Randomised controlled trials Prospective and Retrospective Cohort studies Case series Case reports	Review articles

The data extraction was collected under basic characteristics and outcomes. In each study, mean difference (MD) for continuous outcome and odds ratio (OR) for dichotomous outcome with a 95% confidence interval (CI) was calculated using Review Manager (RevMan) [Computer program, Version 5.3. Copenhagen: The Nordic Cochrane Centre, the Cochrane Collaboration, 2014]. Fixed effect model was used when the heterogeneity was <50%, whereas random effect model was used when the heterogeneity was >50%.

## Results

**Fig. 2:**
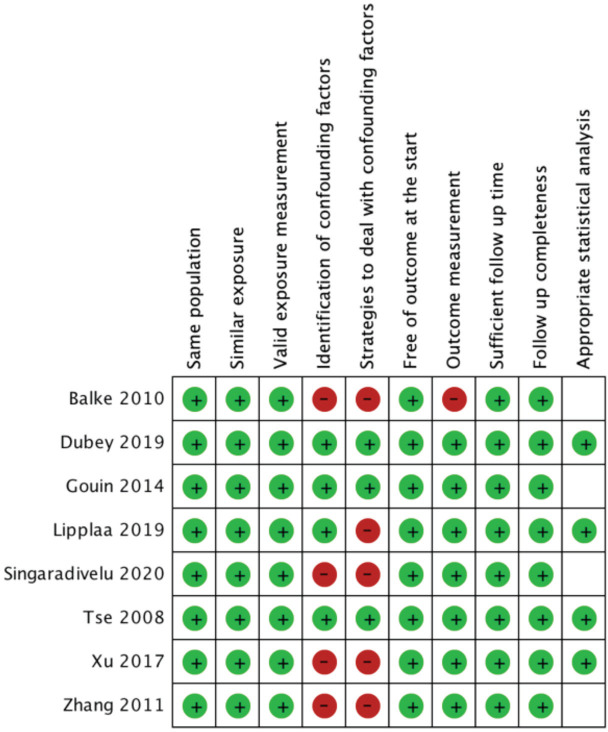
Risk of bias assessment of studies included.

We identified 8 articles from 2008-2020, with a total of 181 cases. Two studies were Level I evidence, two others were Level III, and four others were Level IV (Table II). Critical appraisal of all studies included were conducted based on Joanna Briggs Institute Scoring System, showing no study had more than three invalid parameters (Fig. 2).

**Table II:** Studies included in the analysis

No.	Reference	Journal	Study Design Level	of Evidence
1.	Tse *et al* (2008)^3^	Bone	Case Control Study	Level III
2.	Balke *et al* (2010)^1^	BioMed Central Cancer	Case Series	Level IV
3.	Zhang *et al* (2011)^5^	Spine	Case Reports	Level IV
4.	Gouin *et al* (2014)^7^	European Journal of Cancer	Case Series	Level IV
5.	Xu *et al* (2017)^11^	Journal of Neurosurgery Spine	Cohort Retrospective	Level III
6.	Lipplaa *et al* (2019)^10^	The Oncologist	Randomised Controlled Trial	Level I
7.	Dubey *et al* (2019)^4^	Journal of Clinical Orthopaedics and Trauma	Randomised Controlled Trial	Level I
8.	Singaravadivelu *et al* (2020)^2^	Malaysian Orthopaedic Journal	Case Series	Level IV

From the table of study characteristics, it appeared that GCTB equally affected male and female in control group (30 males vs 27 females) and slightly more female than male in bisphosphonate group (56 males vs 68 females), with the sample age ranging from 19 to 75 years old. The most common locations were distal femur (42 cases) and proximal tibia (33 cases), as well as sacrum (48 cases). Other locations found were proximal femur, proximal tibia, proximal humerus, and distal radius. Appendicular bones were more commonly affected (129 cases) than axial bones (55 cases) (Table III).

**Table III:** Studies included in the analysis

No.	Reference	Sample Size		Age (years)		Sex		Location		Stage	
		Control	BP	Control	BP	Control	BP	Control	BP	Control	BP
1.	Tse et al (2008)^3^	20	24	36(22-52)	36(19-62)	M: 13 F: 7	M: 14 F:10	Prox. humerus: 0 Prox. humerus: 0 Distal radius: 4 Prox. femur: 5 Distal femur: 3 Prox. tibia: 7 Distal tibia: 1	Prox. humerus: 4 Distal radius: 4 Prox. femur: 4 Distal femur: 7 Prox. tibia: 4 Distal tibia: 0	2: 9 3: 11	2:13 3: 11
2.	Balke et al (2010)^1^	-	25	-	15-75	-	M: 9 F: 16	-	Distal femur: 4 Sacrum: 9 Prox. femur: 3 Prox. tibia: 3 Pelvis: 3 Vertebra: 1 Fibula: 2 Prox. humerus: 1 Distal radius: 1	-	1B and 3B Recurrent GCT is also included
3.	Zhang et al (2011)^5^	-	3	-	23, 32, 33	-	F: 3	-	Vertebra T7: 1 Vertebra L5: 1 Sacrum: 1	-	Recurrent GCT in 2 patients.
4.	Gouin et al (2014)^7^	-	20	-	22-70	-	M: 8 F: 12	-	Prox. tibia: 3 Distal tibia: 3 Distal radius: 4 Distal femur: 8 Sacrum: 1 Prox. humerus: 1	-	NA
5.	Xu et al (2017)^11^	16	19	31.9 ± 10.2	33.5 ± 10.8	M: 5 F: 11	M: 7 F: 12	Sacrum region in all patients		2: 2 3: 14	2:6 3: 13
6.	Lipplaa et al (2019)^10^	6	8	45.5 (19-73)	34 (21-55)	M: 4 F: 2	M: 4 F: 4	Distal Femur: 2 Sacrum: 1 Prox. femur: 1 Fibula: 1 Prox. humerus: 1	Distal Femur: 3 Sacrum: 1 Prox. femur: 1 Fibula: 1 Prox. tibia: 1 Spine: 1	Recurrent: 1 High-risk GCTB (Tumours with extension into surrounding soft tissue, intraarticular, pathological fracture, recurrences)	Recurrent: 5 High-risk GCTB (Tumours with extension into surrounding soft tissue, intraarticular, pathological
7.	Dubey et al (2019)^4^	15	15	31.46	32.86	M: 8 F: 7	M: 9 F: 6	Distal femur and Prox. tibia: 10/15 (33.33%) Distal radius: 5/15 (16.66%)	Distal femur and Prox. tibia: 10/15 (33.33%) Distal radius: 5/15 (16.66%)	1: 3/15 (20%) 2: 8/15 (53.3%) 3: 4/15 (26.67%)	1: 3/15 (20%) 2: 7/15 (46.66%) 3: 5/15 (33.33%)
8.	Singaravadivelu et al (2020)^2^	-	10	-	18-39	-	M: 5 F: 5	-	Prox. tibia: 5 Distal femur: 5	-	2: 5 3:3 Recurrent: 2

Most studies administered 4mg of intravenous Zoledronic acid, but other bisphosphonates had also been used, such as Pamidronate, alendronate, clodronate, and sodium ibandronate. Timing of administration differed, with five studies mentioning pre-operative administration and six studies describing post-operative administration. In terms of surgical treatment choice, intralesional curettage and cementation was the most performed procedure (36 cases). Other commonly performed procedures were wide resection and bone grafting (35 cases), intralesional curettage and bone grafting (23 cases), wide resection and cementation (22 cases), and nerve sparing surgery for sacrum GCTB (10 cases). A study by Singaravadivelu *et al* (2020) described 10 cases of GCTB around knee managed by extended curettage and structural support by Fibula Cortical Struts^2^. Phenol and Polymethyl Methacrylate (PMMA) were also used as adjuvant therapies in more than 20 cases, on their own as well as in combination. The follow up period ranged from 3 to 192 months (Table IV).

**Table IV:** Treatment method.

No.	Reference	Type of Bisphosphonate	Administration of Bisphosphonate	Pre/Post-op	Location Detail	Other Treatments	Follow Up
1.	Tse *et al* (2008)^3^	Pamidronate: 7 Zolendronic Acid: 17	Pamidronate: 90mg via 120-min IV Zolendronic Acid: 4mg via 15-min IV	Pre- and Post-op	2 doses before the surgery with each dose at an interval of 3–4 weeks in between After surgical treatment, patients received 3 more doses with each dose at an interval of 3–4 weeks and 3 months of additional oral Clodronate.	Intralesional curettage and cementation: 36 Intralesional curettage and bone grafting: 8 Wide resection and cementation: 22 Wide resection and bone grafting: 22	Control group: 115.4 months (32 to 192 months) Bisphosphonate group: 48 months (24 to 84 months)
2.	Balke *et al* (2010)^1^	Alendronate: 2 Clodronate: 4 Pamidronate: 1 Zoledronic acid: 18	Alendronate: 70mg/week PO Clodronate: 2x800mg/day PO Pamidronate: 90mg/month IV Zoledronic acid: 4mg IV	Pre- and/or Post- op	The timing of administration varies in each patient. Some was given before surgery, some after, and some don’t receive any surgical treatment. Alendronate: for 24-32 months Clodronate: for 12-60 months Pamidronate: Every month Zoledronic acid: up 6 doses	Curettage Embolisation Radiation Ifosfamid, interferon-α, Cyclophosphamide, Cisplatin, Adriamycin	36-64 months
3.	Zhang *et al* (2011)^5^	Sodium Ibandronate	Sodium Ibandronate: 4mg over 120 minutes IV	Post-op	3 doses (2 cases) and 12 doses (1 case) with 4 weeks interval	Intralesional curettage with bone grafting (2 cases), excision, radiotherapy, and thermotherapy (1 case)	2, 4, and 6 years
4.	Gouin *et al* (2014)^7^	Zoledronic acid	Zoledronic acid: 4mg IV	Post-op	5 doses, starting 3–5 days after the operation, then every 3 weeks	Extensive intralesional curettage and filling of the bone cavity using polymethyl methacrylate cement containing antibiotics or bone allograft. A titanium plate was inserted for lower limb when it was considered that there was a high risk of post-operative fracture.	63.6 ± 16 months
5.	Xu *et al* (2017)^11^	Zoledronic acid: 7 Incadronate Disodium: 12	Zoledronic acid: 4mg IV Incadronate Disodium: 10mg IV	Pre- and Post-op	Initial dose was given pre- operatively. 1 dose/ month for 2 years.	Nerve-sparing surgery through posterior approach Pre-operative selective artery embolism (PAE) Cisplatin and methotrexate as local treatment in cases where dura mater was not breached during surgery	Bisphosphonate group: 47.2 ± 9.6 months (36-77 months) Control group: 92.1 ± 43.6 months (21-168 months)
6.	Lipplaa *et al* (2019)^10^	Zoledronic acid	Zoledronic acid: 4mg IV	Post-op	Monthly for 3 months followed by a 3-monthly schedule for up to 1 year after surgery.	Curettage: 3 Curettage with local adjuvants: 9 En bloc resection: 2 Phenol and PMMA: 8 PMMA: 2	Control: 79 (48-97) Bisphosphonate: 97.5 (60-111)
7.	Dubey *et al* (2019)^4^	Zoledronic acid	Zoledronic acid: 5mg IV	Pre-op	3 doses with a gap of 4 weeks between each dose.	Control: 13 patients underwent curettage with bone grafting, 2 patients wide excision followed by reconstruction using endoprosthesis Bisphosphonate: 12 patients underwent extended curettage with bone grafting, 1 patient had wide excision followed by reconstruction using endoprosthesis. 10% phenol, H2O2 was used as adjuvant therapy.	3 months
8.	Singaravadi velu *et al* (2020)^2^	Zoledronic acid	Zoledronic acid: 4mg IV	Pre- and Post-op	1 dose 3 weeks prior to surgery and 2 doses post-operatively (one dose 3 weeks post-surgery and final dose after another 6 weeks)	Extended curettage and structural support by Fibula Cortical Struts.	2.5-3.5 years

Abbreviations - IV: Intravenous, PO: Peroral, PMMA: Polymethyl Metacrylate

**Fig. 3:**
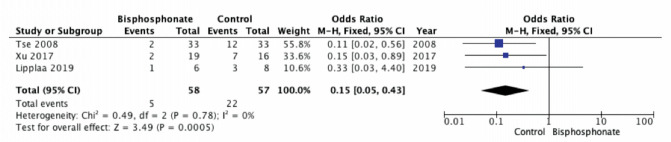
Forest plot for recurrence rate.

**Fig. 4:**
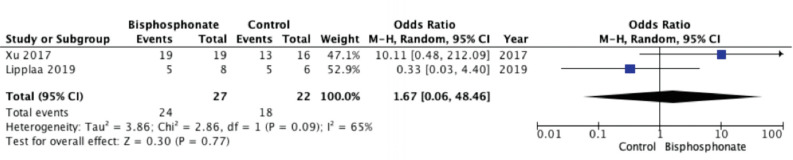
Forest plot for survival rate.

We considered two parameters for the forest plot. The BP group presented lower recurrence rate than control group (three studies; OR 0.15; 95% CI, 0.05 – 0.43; p<0.05; heterogeneity, I2=0%) (Fig. 3). A study by Tse et al (2008), using pre- and post-operative bisphosphonate treatment, contributed the most to the overall final Odds Ratio for recurrence rate (55.8%)^3^. As for survival rate, BP group was comparable to control group (two studies; OR 1.67; 95% CI, 0.06 – 48.46; p=0.77; heterogeneity, I2=65%) (Fig. 4). The metastases rate was low in patients treated with BP, with lung as the most common location. In terms of functional outcome, BP seemed to offer better urinary and bowel function, and most patients were able to return to their pre-surgery functionality. Pain also improved in most patients, with one literature stated that the mean Musculoskeletal Tumour Society (MSTS) Score was 92%. Radiologically, BP was also proven to increase mineralisation and calcification, resulting in stable or decreased size, with better delineated border. Though some side effects have been reported, mostly they were minor and transient, such as fever, headache, or flu-like symptoms (Table V).

**Table V:** Outcomes

No.	Reference	Recurrence Rate	Metastases	Survival Rate	Functional Outcome	Clinical Outcome	Radiological Outcome	Adverse Effect
1.	Tse *et al* (2008)^3^	In stage 2 tumours: Control: 6/20 (30%) Bisphosphonate: 1/24 (4.2%) In stage 3 tumours: Control: 6/13 (46%) Bisphosphonate: 1/9 (9%)	NA	NA	NA	VAS: Improved from 7.7 (5–8) to 3.3 (1–4) Subjective decrease in swelling	Convincing radiological evidence of mineralisation in 14 of the 24 patients (Increased radiodensity, better delineated border)	No untoward effect and no derangements of renal function observed.
2.	Balke *et al* (2010)^1^	Bisphosphonate: 2/25 (8%)	12/25 (48%) (Lung)	24/25 (96%)	NA	VAS: improved pain in 8/25 (32%) patients	Increased calcification in 2/25 patients. Stable size in 11/25 patients, decreased size in 1/25 patients. Slight decrease in the size of lung metastases in 1/25 patients.	No significant side effect
3.	Zhang *et al* (2011)^5^	No further recurrence post bisphosphonate therapy.	NA	3.3 (100%)	NA NA Normal urinary function: Control: 10/16	Pain reduction in all patients	Tumour size reduction and calcification Trabecular bone formation, increased density of lesion	NA
4.	Gouin *et al* (2014)^7^	3/20 (15%)	NA	82±9% at 60 months	Bisphosphonate: 18/19	NA	NA	18/20 (90%) Fever, headache, chest pain, arthralgia, nausea, bone pain, altered taste, urticaria, gastritis.
5.	Xu *et al* (2017)^11^	Control: 7/16 (43.75%) Bisphosphonate: 2/19 (10.53%)	Control: 1/16 Bisphospho- nate: 1/19	Control: 13/16 (Died at 25, 36, and 21 months post- operatively) Bisphos- phonate: 19/19 (100%)	Normal bowel function: Control: 10/16 Bisphosphonate:19/19	Ambulatory function: Control: Normal in all patients Bisphosphonate: 1 patient walks with stick	NA	No significant adverse effect
6.	Lipplaa *et al* (2019)^10^	2 years: Control: 1/6 (17%) Bisphosphonate: 3/8 (38%)	NA	5 years: Control: 83% Bisphospho- nate: 62%	ECOG 0: Control: 2 Bisphosphonate: 3 ECOG 1: Control: 4 Bisphosphonate:5	NA	NA	Fever (13%) Fatigue (25%) Flu-like symptoms (13%) Periodontal disease (13%)
7.	Dubey *et al* (2019)^4^	NA	NA	NA	NA	Pain improved: from 5.33+1.77 to 1.8+0.6 in bisphosphonate group Swelling improved: from 10.56+12.49 to10.73+12.8 (not significant) Mean apoptotic index differs significantly: 41.46 vs. 6.06	NA	No significant adverse effect
8.	Singaravadi velu *et al* (2020)^2^	0%	NA	NA	All patients were able to resume pre-surgery work function	MSTS score: 92% (86.67-96.67%) Knee flexion of 6-120o	Good consolidation of the fibular struts and gradual filling up of the cavity. The joint space was maintained in all cases.	EHL weakness (20%) due to fibular resection, resolved in 6 months.

## Discussion

The locally aggressive nature of GCTB and its ability to metastasize presents a challenge in terms of recurrence and mortality. Surgical resection alone is often insufficient for disease control, and the addition of anti-osteoclastic agent may serve as an alternative to enhance outcomes4. Some adjuvant therapies have been on trial in previous studies, including BP which was already well-known for its efficacy. This systematic review and meta-analysis is the first to summarise recent clinical studies on the subject, focusing on the effectiveness of its regimen and the related outcome.

Bisphosphonates, one of a reliable treatment regimen for osteolytic cancers and metastases, has also been proven to be beneficial in treating osteoclast-mediated bone loss. Bisphosphonates work by binding to hydroxyapatite on the bone surface and inhibit adhesion of osteoclasts to the mineralised bone surface. Bisphosphonates also have a direct effect on stromal cells of GCTs through mevalonate pathway, blocking protein prenylation and promotes the activation. Furthermore, Bisphosphonates inhibit osteoclast-like giant cell formation from immature precursors as well as induces apoptosis in mature osteoclasts. Some literatures have reported the use of bisphosphonates for GCTB with the result of increased mineralisation of lesion as well as replacement of pathological bone lesion into normal bone structure; however, those studies were mostly noncomparative one-arm studies with relatively small amount of samples^5^.

Chang *et al* (2004) in their study proved the inhibitory effect of Bisphosphonates on proliferation and apoptosis of osteoclasts by affecting the osteoprotegerin (OPG)/ Receptor Activator of Nuclear Factor Kβ-Ligand (RANKL) mRNA expression of stromal cells. Furthermore, Zoledronic acid displayed higher efficacy (10–20 times) in apoptosis and decrease in the live-cell rate compared to pamidronate^6^. The ability of BP to lower the amount of osteoclasts and inhibit osteoclastic resorption, especially amino BP, enables it as a potential treatment for GCTB. When applied post-operatively, BP could also eradicate the remaining cells^7^.

Another adjuvant therapy recommended for GCT is Denosumab. As a RANKL inhibitor, Denosumab was proven to be beneficial in tumour growth inhibition and reduced morbidity. A case series study by Goldschlager *et al* (2015) proved that Denosumab demonstrated beneficial radiological and histological response in most patients with spinal GCT^8^. However, in a study by Lau *et al* (2013) comparing Denosumab and Zoledronic Acid, Zoledronic Acid was proven to reduce cell growth, causing apoptosis in most cell lines, and significantly inhibiting mRNA expression of RANKL and Osteoprotegerin. These features were not found in Denosumab, raising a concern that tumour recurrence might happen after drug withdrawal9. Therefore, there were still some controversies in the treatment of choice between the two, as Denosumab failed to prove a permanent apoptotic effect on the neoplastic stromal cell population^6,8^. A study by Gouin *et al* (2014) stated that the local recurrence rate of GCTB in appendicular bones treated by extensive curettage with or without local adjuvant treatment was lower than axial GCTB, where it was around 15% for appendicular bones, 19% for axial bones, and 53% for sacral bones specifically^7^. Regarding the surgical treatment performed, a study by Zhang *et al* (2011) reported cases where interventional blood vessel embolism of the tumour reduced the blood supply, further disturbed the delivery of bisphosphonate to the lesion, and reducing the effectivity of bisphosphonate therapy. Some other adjuvant therapies might also alter the blood supply to GCTB, therefore limiting bisphosphonates’ efficacy, such as radiation and thermotherapy^5^.

In terms of location, sacral GCTB warrants special attention. Despite being one of the most commonly affected bones, the treatment for sacral GCT remains challenging, as sacrificing sacral nerve roots is associated with severe morbidity, such as the disturbance of gait and foot plantar flexion (S1 nerve roots) as well as bowel and bladder dysfunction (S2-3 nerve roots). Even after a successful nerve-sparing surgery, the high recurrence rate (25-35% in most cases and up to 50% in some studies) demands an additional therapy to minimise it, potentially by the use of bisphosphonate therapy^9,10^.

Though some literature supports the efficacy of Bisphosphonates, its use is not without consequences. Bisphosphonate therapy has been reported to relate to some adverse effects in approximately 15% - 30% of cases, though mostly mild such as fever, headache, chest pain, arthralgia, nausea, bone pain, altered taste, urticaria, gastritis, fatigue, flu-like symptoms, and periodontal disease. In more severe cases, osteonecrosis of the jaw could be found in <1% case per year of treatment, however regular dental assessment and avoiding invasive dental procedures are beneficial in preventing this adverse effect^7^.

Another topic still in debate is the timing of bisphosphonate administration. Pre-operative administration of bisphosphonates has been proven to increase peripheral mineralisation, therefore better containing the lesion, more clearly delineating the borders, and making complete removal easier to achieve. However, post-operative administration has also been described in literature, where it was closely related to the recurrence rate. Due to its ability in inducing apoptosis, bisphosphonates can clear the residual microscopic tumour tissue after surgical procedures^2,11^. In our analysis, a study by Tse *et al* (2008)^3^ was proven to have the highest contribution to overall odds ratio in the recurrence rate, leading to a conclusion that pre- and post-operative administration of bisphosphonates in combination might be the most beneficial in minimising the recurrence rate. In terms of survival and metastases rate, bisphosphonates were also comparable to control, making it a considerable choice in the treatment of GCTB, with appropriate dosing and time of administration.

This study has several limitations: (1) Different generations of BPs were used, different surgical techniques (curettage and resection), different protocols (pre- or post-operatively, dose, period of treatment), different adjuvant therapies were applied, and different stages of the disease were treated (primary, recurrent, metastatic). This might contribute to a possible bias of analysis. (2) Due to the scarcity of qualified studies in this field, studies included are mostly of Level IV evidence. However, we have ensured the quality of included studies by using quality and bias assessment. (3) Some studies have short follow up time (3 months), which may contribute to the low rate of adverse effects shown by these studies. Despite these limitations, this study still serves as an important update in the treatment of GCTB, as this is the first meta-analysis study to objectively describe the efficacy of Bisphosphonate therapy. It is hoped that this study will be beneficial in considering adjuvant therapy for GCTB, as well as influential in conducting further well-designed studies with bigger amount of samples.

## Conclusion

Current systematic review and meta-analysis suggest that Bisphosphonate therapy offers satisfactory recurrence rate, functional outcome, clinical outcome, and radiological outcome, as well as comparable survival rate and metastases rate compared to control in patients with GCTB, with minimal adverse effects. The combination of pre- and post-operative administration of bisphosphonates in combination might be the most beneficial in minimising the recurrence rate.
